# 5-Oxoprolinemia in a Patient With Severe Hypothyroidism and Chronic Acetaminophen Use

**DOI:** 10.7759/cureus.34628

**Published:** 2023-02-04

**Authors:** Dariusz Uczkowski, Rani Gundavarapu

**Affiliations:** 1 Internal Medicine, Overlook Medical Center, Summit, USA

**Keywords:** gamma glutamyl cycle, hypothyroidism, acetaminophen, anion gap, 5-oxoprolinemia

## Abstract

5-oxoprolinemia is caused by a defect in the gamma-glutamyl pathway which can present with severe anion gap metabolic acidosis not caused by ketoacidosis, lactic acidosis, methanol/ethylene glycol ingestion, renal failure, ethanol, iron/isoniazid or salicylate ingestion. This case will describe a 59-year-old female presenting with elevated anion gap metabolic acidosis with no discernible classical cause, chronic acetaminophen use, malnourishment, and severe hypothyroidism with 5-oxoprolinemia after extensive investigation of other causes. Treatment involved correcting the acidosis with bicarbonate, IV fluid administration, oral levothyroxine and avoiding further acetaminophen use. The patient’s acidosis resolved soon after and she was counseled on the avoidance of acetaminophen in the future. This case highlights the importance of pharmacologic vigilance with everyday over-the-counter medicines such as acetaminophen and metabolic states such as hypothyroidism which can lead to tumultuous cases of metabolic acidosis. This is the first case in which we know that 5-oxoprolinemia has presented with concomitant severe hypothyroidism. Due to this patient’s course, it may have been the preceding factor for the development of her oxoprolinemia alongside her acetaminophen consumption.

## Introduction

5-oxoprolinemia, also known as pyroglutamic acidosis, is caused by a lack of glutathione produced by the gamma-glutamyl cycle [1,2]. The predilection to this process is related to transient polymorphisms of multiple enzymes in the cycle occurring throughout the patient's life as well as toxic metabolic insult in the adult population [1-3]. The presenting symptoms usually include altered mental status and hyperventilation in the setting of elevated anion gap metabolic acidosis, but patients may also be neurologically intact if presenting early [1-2]. Risk factors for 5-oxoprolinemia include malnourishment, ingestion of certain medications such as acetaminophen, flucloxacillin, netilmicin, vigabatrin, and risk factors like diabetes, liver disease, and pregnancy [1-2]. Leukocyte dysfunction is also prevalent due to granulocytes having decreased glutathione, making it difficult for these patients to mount effective responses to bacterial infections and prolong otherwise normally uncomplicated infections [4-6]. The following report will discuss a 59-year-old female with 5-oxoprolinemia coinciding with chronic acetaminophen use, overt hypothyroidism, urinary tract infection, and malnourishment.

## Case presentation

A 59-year-old female presents with increasing dyspnea over the past few days, frequent urination, decreased oral intake over the past two weeks and diffuse pain after suffering a mechanical fall at home. She additionally reported using two tablets of diphenhydramine-acetaminophen 25-500 mg, three to four times per day over the past two weeks and an herbal medication called Kratom for an unknown period. Past medical history included myocardial infarction status post stenting in 2018, cerebrovascular accident without residual deficits, tobacco use disorder with a 20 pack-year smoking history, chronic back pain and thyroid cancer status post thyroidectomy poorly compliant on levothyroxine for two years. She denied ethanol abuse or other toxic substances. Additional pertinent positives on review of systems included constipation, depression, lower extremity pain/swelling, decreased appetite, dysuria, and lower abdominal pain. Vitals were temperature of 97.6F, respiratory rate 20, blood pressure 131/83, heart rate 85, and oxygen saturation (SpO2) 96% on room air.

Physical exam was remarkable for diffusely dry skin on the upper and lower extremities with flaking and no appreciable rashes or eruptions, dry oral mucosa, resting tremor in the upper extremities bilaterally, no crackles or consolidations in the lungs bilaterally, normal heart rate and rhythm, 1+ non-pitting edema to the level of the shins, alert and oriented x3, lower abdominal tenderness and lower extremity weakness limited by pain with strength 3/5 in the bilateral lower extremities. Chest x-ray was negative for any acute pathology, computed tomography (CT) of the head without contrast was negative for any intracranial processes, CT of the thoracic and lumbosacral spine were without fracture or acute process. Urine analysis revealed protein, blood, nitrites, leukocyte esterase, no crystals and moderate bacteria. Admission labs can be seen in Table 1.

**Table 1:** Labs on admission H: high, L: Low, Na: sodium, K: potassium, Cl: chloride, C02: bicarbonate, BUN: blood urea nitrogen, AST: aspartate aminotransferase, ALT: alanine transaminase, TSH: thyroid stimulating hormone, T4: thyroxine, WBC: white blood cell count, Hgb: hemoglobin

Lab Test	Value	Reference Range
Glucose	149 (H)	70-100 mg/dL
Na	140	135-145 mmol/L
K	3.3	3.2-4.9 mmol/L
Cl	112 (H)	95-110 mmol/L
C02	10 (L)	21-32 mmol/L
Anion gap	18 (corrected with albumin 21.8) (H)	5-15 mmol/L
Albumin	2.5 (L)	3.4-5.0 g/dL
BUN	8	7-18 md/dL
Creatinine	1.19 (H)	0.50-1.00 mg/dL
ethanol	<10.0	<10 mg/dL
AST	44 (H)	15-37 U/L
ALT	49	12-59 U/L
Beta-hydroxybutyrate	1.1	0.2-2.8 mg/dL
Glycohemoglobin A1C	5.3%	4.0-5.6%
TSH	61.9 (H)	0.340-4.820 uIU/ml
T4	Undetectable (L)	0.59-1.80 ng/dL
Acetaminophen level	<10	10-30 ug/mL
Salicylate level	3.7	2.8-20.0 mg/dL
Triglycerides	3,669 (H)	60-150 mg/dL
WBC	13.88 (H)	4.50-11.00/nL
Hgb	10.9 (L)	12.5-16.0 g/dL
Lactic Acid	2.4 (H)	0.4-2.0 mmol/L
Lipase	53	73-393 U/L
Serum osmolality	299	275-300 mOsm/kg
Urine sodium (Na)	33	*mmol reference not established
Urine potassium (K)	35	*mmol reference not established
Urine Chloride (Cl)	65	*mmol reference not established

The patient was treated with ceftriaxone IV for presumptive urinary infection pending cultures, an ampule of bicarb, 2 liters of normal saline boluses and was started on a lactated ringer infusion. Repeat lactic acid was negative after fluid administration the following morning. She was also restarted on levothyroxine via oral administration with 112 mcg daily and initiated on a high-dose statin. Due to the hypertriglyceridemia being present, evaluation for pseudo acidosis was pursued as a cause of elevated anion gap acidosis. An arterial blood gas was obtained which revealed pH 7.24, C02 29mmHg, partial pressure of oxygen (pO2) 77 mmHg, HC03 12 mmol/L, and SpO2 93% on room air. This was indicative of a true acidosis with the bicarbonate being consistent with the comprehensive metabolic panel. Repeat labs were obtained after initial treatment as well as a 5-oxoproline level due to all other classical causes of elevated anion gap metabolic acidosis being effectively ruled out (Table 2).

**Table 2:** Labs after initial treatment H: High, L: Low, C02: bicarbonate, TSH: thyroid stimulating hormone, T4: thyroxine

Lab Test	Value	Reference Range
Anion Gap	13	5-15 mmol/L
C02	17 (L)	21-32 mmol/L
Triglycerides	1,152 (H)	60-150 mg/dL
TSH	30 (H)	0.340-4.820 uIU/ml
T4	0.29 (L)	0.59-1.80 ng/dL
5-oxoproline	8,844 (H)	<62 mmol/mol creatinine
Creatinine	0.83	0.5-1.00 mg/dL
Lactic acid	0.4	0.4-2.0 mmol/L

The anion gap subsequently closed soon after, the bicarbonate levels improved, triglycerides trended down, thyroid stimulating hormone (TSH) and thyroxine (T4) began to improve and the 5-oxoproline level returned elevated. The patient continued ceftriaxone for two days and was transitioned to oral cephalexin on day three for the rest of a five-day course. As hospitalization progressed, her metabolic acidosis resolved with the anion gap remaining closed on continuous fluid administration, low-fat diet, levothyroxine administration and avoidance of acetaminophen. Repeat 5-oxoproline level one week later was normal.

## Discussion

Multiple factors such as acetaminophen use, malnutrition, and hypothyroidism could have led to alterations in the enzymatic processes of the gamma-glutamyl pathway leading this patient to develop a 5-oxoprolinemia elevated anion gap metabolic acidosis. However, the incidence of 5-oxoprolinemia is frequently described in the literature in pediatric populations rather than adults [1]. An autosomal recessive form of glutathione synthetase deficiency that predisposes pediatric populations to develop elevated anion gap metabolic acidoses with 5-oxoprolinemia typically begins in the neonatal period and has devastating outcomes [1]. Cases present with severe hemolytic anemia, neurological deficits, early morbidity, and mortality; however, given that this patient was an adult on presentation and did not have any prior history of 5-oxoprolinemia, the likelihood of having an autosomal recessive pattern could not be the case [1]. With this being said, chronic acetaminophen use in adult females causing 5-oxoprolinemia has been a reported etiology in case reports, basic science text books and nephrology societies [2]. The transient alterations in the enzymatic scheme of the gamma-glutamyl pathway will be discussed further as well as the individual insults to the cycle that were present during this patient’s hospital course.

The pharmacology of acetaminophen is known to deplete glutathione stores in the liver, thus significantly impairing the liver’s ability to effectively participate in the multiple oxidative reactions necessary to clear toxins and metabolites from our organism [2]. When put in conjunction with individuals who have poorly understood transient polymorphisms in the enzyme glutathione synthetase, the risk for developing 5-oxoprolinemia increases and can be fatal if not recognized [2]. This acidotic process begins with a glutathione-depleted state, due in part to acetaminophen in this case, which induces the enzyme gamma-glutamyl cyclotransferase to produce 5-oxoproline and inhibit the enzyme 5-oxprolinase from breaking down 5-oxoproline, resulting in an elevated anion gap metabolic acidosis secondary to the build-up of 5-oxoproline [2]. 

**Figure 1:**
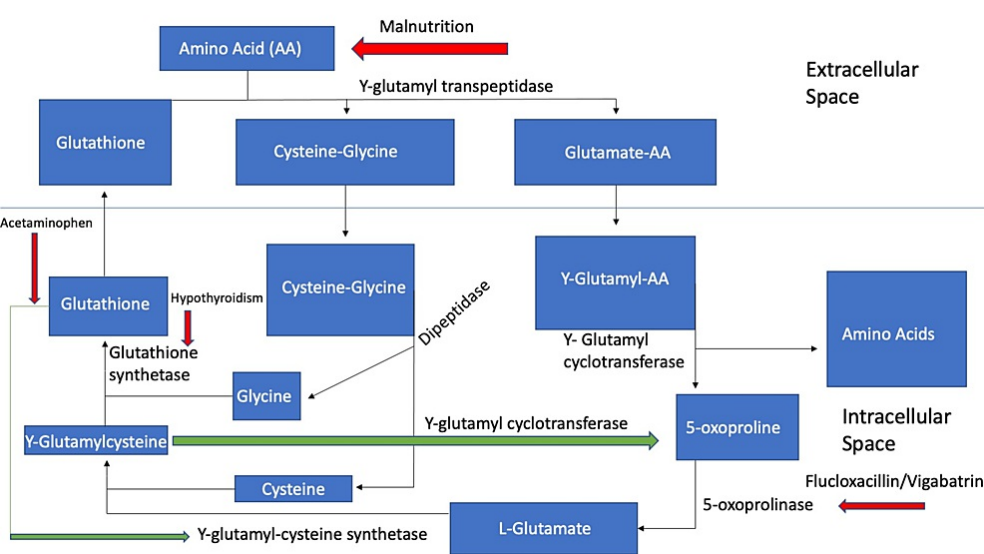
Gamma-Glutamyl Cycle Red highlights: steps that are inhibited by each pathologic process (acetaminophen, hypothyroidism, malnourishment), Green highlights: negative feedback processes, y: gamma, AA: amino acid Image Credits: Dariusz Uczkowski

Severe hypothyroidism has also been associated with decreased activity of the enzyme riboflavin kinase, which is an enzyme necessary in the formation of the cofactors flavin mononucleotide (FMN) and flavin adenine dinucleotide (FAD) and is responsible for several steps in the gamma-glutamyl cycle [2]. The importance of these cofactors is illustrated in the reaction that catalyzes oxidized glutathione to reduced glutathione. In the absence of reduced glutathione being readily available to provide feedback inhibition in the gamma-glutamyl pathway, 5-oxoproline begins to build in the body via increased activity of glutamyl cyclotransferase (Figure 1) [2].

An additional factor in this patient’s presentation to consider was the herbal medication Kratom, which has not previously been associated with 5-oxoprolinemia but was a reported medication that the patient was taking prior to arrival. A literature review of Kratom has not revealed an association with 5-oxoprolinemia, however, reports of toxic liver injury with classically elevated aspartate aminotransferase (AST) and alanine aminotransferase (ALT) have been observed and have been managed with abstinence from the substance in tandem with excellent supportive care [3]. Our patient was not consistent with the clinical picture of hepatic injury associated with Kratom as only a slight transaminitis could be observed and therefore would not coincide with the typical presentation of Kratom toxicity [3].

Interestingly, 5-oxoprolinemia can also cause leukocyte dysfunction, which likely contributed to this patient’s otherwise uncomplicated urinary tract infection course due to glutathione levels being less readily available to keep up with the granulocyte demand [4]. This is primarily due to the increased demand for glutathione by granulocytes and having less available glutathione due to the shunting of 5-oxoprolinemia production [4]. As a result, there will be a typical early respiratory burst, but the continuous production of toxic oxygen products normally detoxified by glutathione’s reduction of hydrogen peroxide results in auto oxidative damage and defective microbicidal activity [4]. Malnourishment of amino acids, vitamins and trace minerals can also contribute to the dysfunction of the gamma glutamyl cycle as well as to granulocyte function [4,5]. This risk factor is classically described in other case reports as an etiology for developing 5-oxoprolinemia and would have contributed to decreased availability of glutathione via decreased amino acid intake and reduced glutathione production also illustrated in Figure 1.

A helpful mnemonic called “GOLDMARK” can be used when evaluating causes of elevated anion gap metabolic acidoses such as in this case [5]. These include glycols (ethylene and propylene), oxoproline, L-lactate, D-lactate, methanol, aspirin, renal failure and ketoacidosis [5]. Other notable risk factors for the development of 5-oxoprolinemia include ingestion of certain medications such as flucloxacillin, netilmicin, vigabatrin, and medical factors like diabetes, liver disease, and pregnancy [6]. Regarding the treatment of 5-oxoprolinemia, there are currently no societal guidelines given the rarity of the condition, however, proposed recommendations from case reports and literature reviews are to avoid causative substances/processes such as acetaminophen and malnutrition [6]. Early fluid administration and possible N-acetylcysteine has been used in severe cases with refractory improvement to the removal of causative agents and rapid decline. Still, recommendations for N-acetylcysteine are on a case-by-case basis [6]. In some cases, hypertriglyceridemia can be the cause of diabetic ketoacidosis which causes a high anion gap metabolic acidosis [7]. Serum osmolarity, beta hydroxybutyrate and glucose levels will be high in those cases [7]. This patient, however, did not have derangements in those parameters and therefore her hypertriglyericedemia, although high, was not the cause of her high anion gap metabolic acidosis as she was also not in ketoacidosis given her negative beta hydroxybutyrate levels [7].

## Conclusions

In conclusion, it is crucial to highlight the need for vigilance regarding uncommon causes of elevated anion gap metabolic acidoses such as 5-oxoprolinemia, which can coincide with states such as severe hypothyroidism and over-the-counter medicines such as acetaminophen. Severe hypothyroidism has not been recognized previously in the literature as a cause of 5-oxoprolinemia, but this case highlights the importance of severely compromised metabolic states like severe hypothyroidism, which can predispose to this pathology developing. Therefore, this differential should be high when a patient presents with elevated anion gap metabolic acidosis as it may lead to decreased time to diagnosis and treatment for these patients.
